# Genome-Wide Analysis of *DA1-Like* Genes in *Gossypium* and Functional Characterization of *GhDA1-1A* Controlling Seed Size

**DOI:** 10.3389/fpls.2021.647091

**Published:** 2021-05-20

**Authors:** Shuxian Yang, Li Huang, Jikun Song, Lisen Liu, Yingying Bian, Bing Jia, Luyao Wu, Yue Xin, Man Wu, Jinfa Zhang, Jiwen Yu, Xinshan Zang

**Affiliations:** ^1^Zhengzhou Research Base, State Key Laboratory of Cotton Biology, Zhengzhou University, Zhengzhou, China; ^2^State Key Laboratory of Cotton Biology, Institute of Cotton Research of Chinese Academy of Agricultural Sciences, Key Laboratory of Cotton Genetic Improvement, Ministry of Agriculture and Rural Affairs, Anyang, China; ^3^Department of Plant and Environmental Sciences, New Mexico State University, Las Cruces, NM, United States

**Keywords:** cotton, seed, *DA1-like*, expression pattern, *GhDA1-1A*

## Abstract

Cotton (*Gossypium* spp.) is an economically important crop grown for natural fiber and seed oil production. *DA1* is a ubiquitin receptor that determines final seed and organ size by restricting the period of cell proliferation. In the present study, we identified 7 *DA1-like* genes each in cultivated tetraploid (AADD) *G. hirsutum* and *G. barbadense*, and 4 and 3 *DA1-like* genes in their ancestral diploid *G. arboreum* (A2A2) and *G. raimondii* (D5D5), respectively. The 7 *GhDA1* genes were confirmed to be distributed on four At and three Dt subgenome chromosomes in *G. hirsutum*. *GhDA1-1A* showed a high sequence similarity to *AtDA1* in *Arabidopsis*, and they possessed the same functional domains, suggesting conserved functions. The overexpression of *GhDA1-1A*^*R*301*K*^ in *Arabidopsis* significantly increased seed size and seed weight, indicating that *GhDA1-1A* is a promising target for cotton improvement. This study provides information on the molecular evolutionary properties of *DA1-like* genes in cotton, which will be useful for the genetic improvement of cotton.

## Introduction

Organ size is one of the most important features and is regulated by complex developmental processes involving both internal and external signals (Cai et al., 2020). Seeds represent the core of plant life cycle traits and are involved in the mechanisms of plant diffusion, germination, seedling survival and overall reproductive success (Cardinal-McTeague et al., 2019; Keren et al., 2020). In contrast to the reproductive advantage of small-seeded species, the key advantage of larger seeds appears to be their tolerance to abiotic stresses such as shade or drought, and seed size is also an important agronomic trait that greatly affects crop yield (Liu et al., 2020b). The signaling pathways that affect endosperm and/or maternal tissue growth to determine seed size include the *HAIKU1* (IKU), ubiquitin-proteasome, G-protein signaling, and mitogen-activated protein kinase, plant hormone, and transcription regulator pathways (Li and Li, 2016).

Cotton is an economically important crop. In cotton breeding, focusing on a higher lint percentage (a ratio between lint weight and total seed cotton weight from seed and lint) inadvertently leads to the reduction in seed size or weight (i.e., the seed index, or the weight in g of 100 cotton seeds), which is an indicator of the quality of seeds (Zhao et al., 2015a). Generally, cottonseeds with larger volume and mass tend to content more storage material and have higher vigor (Zhao et al., 2019). Previous studies have shown that plants with large seeds exhibit better traits than those with small seeds on the basis of testing the effect of seed size on cotton seedling growth. Large seeds exhibit more nutrient accumulation than small seeds, which may affect seed germination and even the growth and development of plants (Wang et al., 2008).

There are many factors affecting the size of a seed, including genetic factors, environment (including pests and diseases) and genotype-by-environment interactions (Wang et al., 2016). In recent years, the completion of plant genome sequencing and the construction of plant transcription factor databases have greatly advanced research progress related to transcription factors involved in seed development and their molecular regulation mechanisms (Wang et al., 2013). It has been shown that multiple components of the ubiquitin pathway are involved in the regulation of seed size: for example, E3 ubiquitin ligases, the proteasome, and ubiquitination modification play important roles in regulating seed size. Several genes related to plant organ size have been cloned and functionally verified, such as *EBPI* (Horváth et al., 2006), *DA1* (Li et al., 2008) and *DA2* (Xia et al., 2013). There are three different protein domains in AtDA1, including two UIMs proximal to the N-terminus, one zinc-binding LIM domain and one DA1-like functional domain next to the C-terminus (Peng et al., 2015). The *DA1* genes of *Arabidopsis thaliana* encode a ubiquitin receptor. When the conserved arginine (R) at the 358th position in the AtDA1 protein sequence is mutated to lysine (K), the resulted mutant produces larger seeds than wild-type plants, indicating that the *DA1* gene negatively regulates both seed and tissue size (Li et al., 2008). The overexpression of *AtDA1*^*R*358*K*^ can increase the rapeseed yield in *Brassica napus* (Wang et al., 2017). In addition, the overexpression of the mutant *ZmDA1* (*Zmda1*) or *ZmDAR1* (*Zmdar1*) gene improves sugar import in sink organs and starch synthesis in maize kernels (Xie et al., 2018).

However, DA1-like proteins without conserved mutation may play different roles in different plant species. Different cis-acting regulatory elements in the promoter sequences of *Arabidopsis thaliana* and rice respond to different hormones (such as abscisic acid and salicylic acid) and stress signals (such as heat stress and drought stress) (Li et al., 2009). As a receptor for E3 ubiquitin ligase, *DA1-like* genes also play important roles in regulating ABA signaling pathways to participate in drought stress (Li et al., 2008). In *Glycine soja*, constitutive *GsoDA1* expression can improve salt resistance with no effect on seed size (Zhao et al., 2015b). The overexpression of TaDA1 decreased the size and weight, while the downregulation of *TaDA1* might be effective in improving grain yields in wheat (Liu et al., 2020a).

Till now, there is no report whether *DA1-like* genes from cotton regulate seed size. In the present study, sequence characteristics and expression patterns of *DA1-like* genes were analyzed in cotton. *GhDA1-1A* is the homologous gene of *AtDA1* in cotton. Previous studies had demonstrated that seed size of At*da1-1* mutant increased (Li et al., 2008). We wanted to know whether over-expression of mutated *GhDA1-1A* would have a similar phenotype. Then, *GhDA1-1A*^*R*301*K*^ sequence was designed containing a single-nucleotide G-to-A transition at 902nd nucleotide site of *GhDA1-1A* (GH_A01G1154), which was predicted to cause an arginine-to-lysine change at the 301st amino acid site. *GhDA1-1A*^*R*301*K*^ was transformed into Arabidopsis thaliana ecotype Col-0. The relationship between *GhDA1-1A*^*R*301*K*^ and seed size was elaborated.

## Materials and Methods

### Sequence Retrieval and Identification of *DA1-Like* Genes in *Gossypium*

The DA1 genome sequences and protein sequences of *Arabidopsis* and *Glycine max* were retrieved from The *Arabidopsis* Information Resource (TAIR release 10)^1^ and SoyBase^2^, respectively. At the CottonGEN website^3^, we downloaded the genome sequences of *G. arboreum* (A2, CRI_V1.0) (Du et al., 2018), *G. raimondii* (D5, JGI v2_a2.1) (Paterson et al., 2012), *G. hirsutum acc*. TM-1 (AD1, ZJU) (Hu et al., 2019), and *G. barbadense acc*.3-79 (AD2, ZJU) (Hu et al., 2019). The candidate DA1 protein sequences were used as the query sequences, and BlastP (E-value = 10 × 10^–5^) searches were performed in the above genome databases. The default parameter settings were used. Then, the candidate sequences were submitted to Pfam^4^ (Finn et al., 2014) and further verified in the SMART^5^ (Letunic et al., 2015) database to determine whether the candidate sequence contained one zinc-binding LIM domain and one DA1-like functional domain next to the C-terminus. Multiple sequence alignments of all *DA1* full-length protein sequences were performed using Clustal X2.0 software (Larkin et al., 2007) with the default values. Subsequently, the neighbor-joining (NJ) method was employed to construct phylogenetic trees by using MEGA v7.0 software (Tamura et al., 2013) with the pairwise deletion option, Poisson correction model and uniform rates. The statistical reliability of the phylogenetic tree was evaluated using the bootstrap method with 1000 repeats. Furthermore, the theoretical molecular weight (MW) and isoelectric point (pI) of the DA1-like proteins were predicted using the online ExPASy tool^6^ (Bjellqvist et al., 1994).

### Chromosomal Location

All *DA1-like* genes of *G. raimondii*, *G. arboreum*, *G. hirsutum*, and *G. barbadense* were mapped on the corresponding chromosomes according to their positional information provided in the genome annotation document. The chromosome location of the cotton *DA1-like* genes was illustrated with MapChart v2.2 software (Voorrips, 2002).

### Genetic Structure Analysis and Protein Domain Detection

Tbtools software (Chen et al., 2020) was used to predict *DA1-like* gene structure. The NCBI database^7^ was used for the identification of DA1-like protein domains.

### Plant Materials and Growth Conditions

Upland cotton TM-1 was used for gene cloning and spatiotemporal quantitative real-time PCR (qRT-PCR) analysis and was grown at Anyang (AY), Henan, China. Roots, stems and leaves were collected at the seedling stage, and fibre and ovule samples were collected at 0, 5, 10, 20 and 30 days post-anthesis (DPA) for RNA extraction. Each experiment was independently repeated in triplicate. *Arabidopsis* thaliana ecotype Columbia-0 (Col-0) was used as the wild-type line. The RNA-seq data of *G. hirsutum* acc. TM-1 (Hu et al., 2019) were used to identify the expression levels of GhDA1-like genes. The expression profiles of AtDA1 and AtDAR1-7 genes were extracted from the *Arabidopsis* eFP Browser database^8^ were used to identify the expression levels of AtDA1 and AtDAR1-7 genes. Seeds were surface-sterilized with 10% (v/v) household bleach for 10 min, washed at least three times with sterile water, stratified at 4°C for 3 days in the dark, dispersed on Murashige and Skoog (MS) medium with 0.88% agar, and then grown at 22°C. Plants were grown under long-day conditions (16-h light/8-h dark) at 22°C.

### Constructs and Transformation

The complete coding sequence of *GhDA1-1A* was amplified with gene-specific primers (Supplementary Table 1). *GhDA1-1A*^*R*301*K*^ DNA sequencing revealed a single-nucleotide G-to-A transition in the *GhDA1-1A* gene (GH_A01G1154), which was predicted to cause an arginine-to-lysine change at the 301^*st*^ amino acid site. GhDA1-1A-F and mGhDA1-1Ar as well as mGhDA1-1Af and GhDA1-1A-R bridge PCR primer pairs were used in the first PCR amplification to introduce a G to A single-base mutant in two fragments. GhDA1-1A-F and GhDA1-1A-R were used to join the two fragments together. The complete coding sequence of *GhDA2* was amplified with gene-specific primers (Supplementary Table 1).

The *35S:GhDA1-1A*^*R*301*K*^ construct was generated using a PCR-based system. The specific primers used to produce the *35S:GhDA1-1A*^*R*301*K*^ construct were 35S:GhDA1-1A^*R*301*K*^-F and 35S:GhDA1-1A^*R*301*K*^-R (Supplementary Table 1). The PCR products were subcloned into a pBI121 vector digested with *Bam*HI and *Sac*I using the ClonExpress^®^ II One Step Cloning Kit (Vazyme, Nanjing, China). The 35S:*GhDA1-1* plasmid was introduced into Col-0 plants using *Agrobacterium* GV3101, and transformants were selected on medium containing kanamycin (50 mg/L). The progeny of the transformants showed approximately 3:1 segregation of live and dead phenotypes, and homozygous lines of the T3 generation were used for further analysis.

### Subcellular Localization Analysis

The CDS of *GhDA1-1A* (1,431bp) and *GhDA2* (1,272 bp) was cloned into the *Kpn*I and *Sma*I sites of the 35S-GFP vector to generate 35S-GhDA1-1-GFP and 35S-GhDA2-GFP with a ClonExpress II One Step Cloning Kit (Vazyme, C112-01). The 35S-GhDA1-1-GFP and 35S-GhDA2-GFP construct was introduced into tobacco (*N. benthamiana*) leaves, respectively. GFP fluorescence in tobacco leaves were observed by confocal microscopy.

### Bimolecular Fluorescence Complementation Assays

The CDS of GhDA1-1A was cloned into the *Bam*HI and *Sal*I sites of the *pSPYNE* vector and the CDS of *GhDA2* was cloned into the *Bam*HI and *Sal*I sites of the *pSPYCE* vector with a ClonExpress II One Step Cloning Kit (Vazyme, C112-01), respectively. The constructs were transferred into *Agrobacterium* GV3101 cells. *Agrobacterium* cells were grown in LB medium containing 1% (m/v) peptone, 0.5% (m/v) yeast extract, and 1% (m/v) NaCl (pH 7) at 28°C to an OD_600_ of 1.2. The bacteria were pelleted and resuspended at a concentration corresponding to an OD_600_ of 1.2 in a solution containing 10mM MES (pH 5.8), 10mM MgCl2, and 150mM acetosyringone. Different combinations of pSPYNE-GhDA1-1A/pSPYCE-GhDA2, pSPYNE/pSPYCE-GhDA2, pSPYNE-GhDA1-1A/pSPYCE, and pSPYNE/pSPYCE were infiltrated into *N. benthamiana* leaves. The YFP fluorescence was detected 2 days after infiltration by confocal microscopy.

### Luciferase Complementation Imaging Assay

The CDS of *GhDA1-1A* was cloned into the *Sac*I and *Sal*I sites of the pCAMBIA1300-nLUC vector with a ClonExpress II One Step Cloning Kit (Vazyme, C112-01). The CDS of *GhDA2* was cloned into the *Kpn*I and *Sal*I sites of the pCAMBIA1300-cLUC vector with a ClonExpress II One Step Cloning Kit (Vazyme, C112-01). The constructs were transferred into *Agrobacterium* GV3101 cells. *Agrobacterium* cells were grown in LB medium containing 1% (m/v) peptone, 0.5% (m/v) yeast extract, and 1% (m/v) NaCl (pH 7) at 28°C to an OD_600_ of 1.2. The bacteria were pelleted and resuspended at a concentration corresponding to an OD_600_ of 0.6 in a solution containing 10mM MES (pH 5.8), 10mM MgCl_2_, and 150mM acetosyringone. Different combinations of *GhDA1-1A-nLuc/c-Luc-GhDA2*, *nLuc/c-Luc-GhDA2*, and *GhDA1-1A-nLuc/c-Luc* were introduced into *N. benthamiana* leaves by *Agrobacterium tumefaciens*-mediated transformation. Luciferase activity was detected 2 days after infiltration. Luciferin (Promega, e1601) at a 1 mM concentration was sprayed onto leaves, and the materials were kept in the dark for 10 min. Images were obtained with a charge-coupled device (CCD) imaging apparatus (Tanon-5200 Multl, Shanghai China).

## Results

### Genome-Wide Identification and Phylogenetic Analysis of *DA1-Like* Genes in *Gossypium*

To identify all the DA1-like proteins in cotton, BLASTP searches were performed against the diploid cotton (*G. raimondii* and *G. arboreum*) and tetraploid cotton (*G. hirsutum* and *G. barbadense*) protein databases using the AtDA1 and AtDAR1-7 protein sequences of *Arabidopsis* as queries. The candidate genes were further subjected to analysis in the NCBI database to identify their protein domains. After a strict two-step selection process, 4 deduced DA1-like genes were identified in *G. arboreum*, along with 3 in *G. raimondii*, 7 in *G. barbadense*, and 7 in *G. hirsutum*. More information about *DA1-like* genes, such as identifiers and predicted properties of DA1-like proteins, is listed in Table 1.

**TABLE 1:** Characteristics of *DA1-like* genes and predicted properties of DA1-like proteins.

Family name	Gene name	Gene identifier	Chromosomal localization	pI	MW (KD)	Size (AA)
group1	Ga01G1461	*GaDA1-1*	A01	5.64	54.11	472
	Ga05G2088	*GaDA1-2*	A05	6.43	58.73	511
	Ga12G2371	*GaDA1-4*	A12	5.31	64.54	569
	Gorai.009G205600	*GrDA1-2*	D05	6.25	57.49	499
	Gorai.008G070600	*GrDA1-4*	D12	5.2	61.96	548
	GH_A01G1154	*GhDA1-1A*	A01	5.64	54.72	476
	GH_A05G1969	*GhDA1-2A*	A05	6.47	54.93	477
	GH_D05G2007	*GhDA1-2D*	D05	6.39	54.96	477
	GH_A12G0677	*GhDA1-4A*	A12	5.18	62.22	549
	GH_D12G0689	*GhDA1-4D*	D12	5.38	60.76	537
	GB_A01G1170	*GbDA1-1A*	A01	5.64	54.72	476
	GB_A05G1997	*GbDA1-2A*	A05	6.32	56.82	494
	GB_D05G2023	*GbDA1-2D*	D05	6.39	55.05	478
	GB_A12G0703	*GbDA1-4A*	A12	5.14	62.15	549
	GB_D12G0687	*GbDA1-4D*	D12	5.46	60.79	537
group2	Ga10G0237	*GaDA1-3*	A10	8.81	56.54	501
	Gorai.011G270900	*GrDA1-3*	D10	8.37	58.13	519
	GH_A10G2480	*GhDA1-3A*	A10	8.37	58.21	520
	GH_D10G2599	*GhDA1-3D*	D10	8.37	58.56	522
	GB_A10G2653	*GbDA1-3A*	A10	8.29	54.20	482
	GB_D10G2613	*GbDA1-3D*	D10	8.37	58.77	525

**FIGURE 1:**
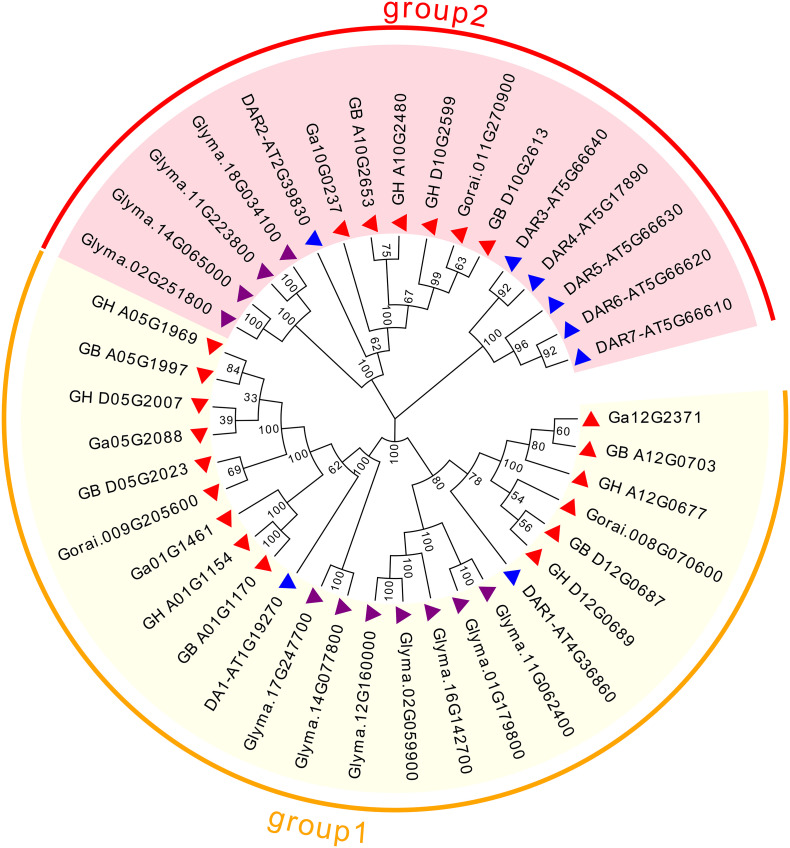
Phylogenetic relationships of *DA1-like* genes from *Gossypium, Arabidopsis*, and soybean. Phylogenetic analysis was performed using the neighbor-joining method with 1,000 replicates. The *DA1-like* genes from *Gossypium, Arabidopsis*, and soybean are marked with red, blue, and purple triangles, respectively. Each group is indicated with a specific color.

To assess the phylogenetic relationships of *DA1-like* genes among four cotton species, *Arabidopsis* and soybean, a comprehensive phylogenetic tree was constructed using the NJ method (Figure 1). In accordance with previous studies (Zhao et al., 2015b), the *DA1-like* genes could be divided into two groups. There were 15 members in *DA1-like* group 1: 5 from *G. hirsutum*, 5 from *G. barbadense*, 3 from *G. arboreum*, and 2 from *G. raimondii*. In addition, *DA1-like* group 2 consisted of 6 members: 2, 2, 1, and 1 from *G. hirsutum*, *G. barbadense*, *G. arboretum*, and *G. raimondii*, respectively.

### Chromosomal Distribution of *DA1-Like* Genes

The mapping of 21 *DA1-like* genes to chromosomes based on the available genomic information on the four cotton species revealed that all the *DA1-like* genes were evenly distributed on chromosomes. In the *G. arboreum* genome, 4 *GaDA1s* were distributed on four chromosomes (A01, A05, A10, and A12) (Table 1 and Supplementary Figure 1A). Three *GrDA1* genes were mapped to 3 chromosomes of *G. raimondii*: chromosome 09 (D05), chromosome 11 (D10), and chromosome 08 (D12) (Table 1 and Supplementary Figure 1B). In the *G. hirsutum* genome, we found that four *GhDA1s* were located on At subgenome chromosomes (A01, A05, A10, and A12), while three *GhDA1s* genes were located on three Dt subgenome chromosomes (D05, D10, and D12) (Table 1 and Supplementary Figure 1C). In the *G. barbadense* genome, we also found that four *GbDA1s* were located on the four At subgenome chromosomes (A01, A05, A10, and A12). The other three *GbDA1s* were located on Dt subgenome chromosomes (D05, D10, and D12) (Table 1 and Supplementary Figure 1D). We speculate that *DA1-like* genes were conserved during diploid to tetraploid evolution.

### Gene Structure and Protein Domain Analyses of *DA1-Like* Genes

**FIGURE 2:**
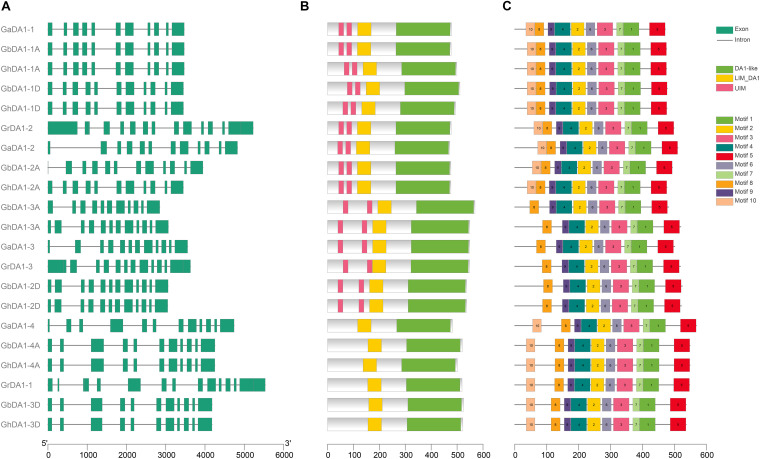
Gene structure and protein domain analyses of *DA1-like* genes in *Gossypium*. **(A)** The exon-intron structure of *DA1-like* genes in *G. raimondii, G*. *arboreum, G. hirsutum*, and *G. barbadense*. **(B)** DA1-like protein domain prediction. **(C)** The motifs of DA1-like genes in *G. raimondii, G*. *arboreum, G. hirsutum*, and *G. barbadense*, respectively.

The analysis of gene structure is a very effective method for determining gene function and can reflect the phylogenetic relationships among *DA1-like* genes. By comparing the GFF3 files of each *DA1-like* gene family member in *G. hirsutum*, *G. barbadense*, *G. raimondii*, and *G. arboreum* with their corresponding coding sequences, we evaluated the gene structure of *DA1-like* gene family members (Figure 2A). A common feature that can be observed is that *DA1-like* genes may contain more than ten exons: eleven *DA1-like* genes contain 11 exons, eight genes contain 12 exons and two genes contain 13 exons (Figure 2A). To better understand the similarity and diversity of DA1 proteins, their putative protein domains were predicted using the NCBI database. Previous studies have shown that UIM domains are absent in some DA1-like proteins (Li et al., 2008). Our results showed that most of the DA1-like proteins contained two UIM domains, one LIM domain, and one conserved DA1-like protein domain at the C-terminus. However, GrDA1-1, GhDA1-3D, GbDA1-3D, GaDA1-4, GhDA1-4A, and GbDA1-4A exhibited only one LIM domain and one DA1-like protein domain (Figure 2B). In addition, ten different conserved motifs were indicated by the MEME online program^9^ (Bailey and Elkan, 1994). It was demonstrated that most of these motif sare conserved in DA1-like proteins, except in *G*bDA1-3A, GhDA1-3A, GaDA1-3, GrDA1-3, GbDA1-2D, and GhDA1-2D, in which motif-10 was absent. It remains to be determined whether the lack of motif-10 alters protein function (Figure 2C).

### Tissue-Specific Expression Profiles of GhDA1 Genes

**FIGURE 3:**
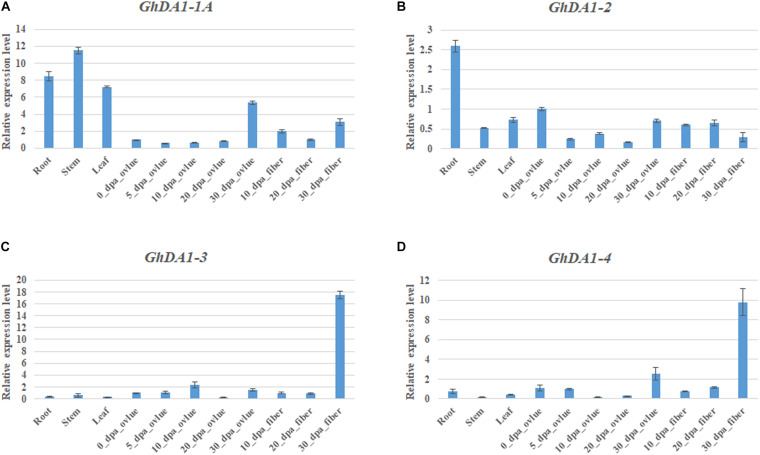
Tissue-specific expression profiles of *GhDA1s* in different tissues of *G. hirsutum* accession TM-1. **(A)** Relative expression level of *GhDA1-1A*. **(B)** Relative expression level of *GhDA1-2A* and *GhDA1-2D*. **(C)** Relative expression level of *GhDA1-3A* and *GhDA1-3D*. **(D)** Relative expression level of *GhDA1-4A* and *GhDA1-4D*. The ΔC_*t*_ value of *GhDA1* in 0-DPA-ovules was set as the control. The data presented are the means ± SD of three replicates.

To reveal the tissue-specific expression profiles of *DA1-like* genes in cotton and *Arabidopsis*, published TM-1 expression data (Hu et al., 2019) and the public *Arabidopsis* expression data (See Text Footnote 8) were used for analysis (Supplementary Figure 2A,B). *GhDA1-like* genes exhibited different expression patterns in different tissues of TM-1 as that in *Arabidopsis*, indicating that *GhDA1-like genes* have multiple biological functions in cotton growth and development. qRT-PCR results also showed that *GhDA1-1A* was highly expressed in roots, stems, and leaves but presented almost no expression in the early stage of ovule development (Figure 3A). *GhDA1-2* (including the expression of *GhDA1-2A* and *GhDA1-2D*) was most highly expressed in roots (Figure 3B). *GhDA1-3* (including the expression of *GhDA1-3A* and *GhDA1-3D*) and *GhDA1-4* (including the expression of *GhDA1-4A* and *GhDA1-4D*) expression was highest in 30 DPA fibers, suggesting the involvement of these genes in the late period of cotton fiber development (Figures 3C,D).

*GhDA1-1A* and *AtDA1* showed similar expression patterns and were widely expressed, indicating that *GhDA1-1A* has similar biological functions during cotton growth and development.

### Generation of *GhDA1-1A*^*R*301*K*^-Overexpressing *Arabidopsis* Lines

**FIGURE 4:**
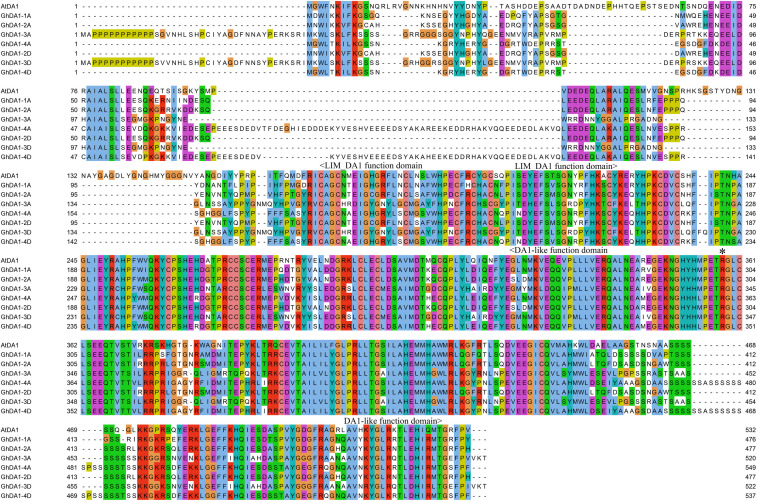
Multiple sequence alignments of amino acid sequences.

AtDA1, GhDA1-1A, GhDA1-2A, GhDA1-3A, GhDA1-4A, GhDA1-2D, GhDA1-3D, and GhDA1-4D contain 532, 476, 477, 520, 549, 477, 522, and 537 amino acids, respectively, and they share 44.31% to 63.79% identity, indicating the high conservation of these homologs. The amino acid sequence of GhDA1-1A showed the closest similarity to that of AtDA1 and contained the same functional domain (Figure 4). The overexpression of *AtDA1*^*R*358*K*^ results in large seeds and organs (Li et al., 2008; Weng et al., 2008), therefore, we were interested in whether *GhDA1-1A* could exhibit similar functions. Hence, *GhDA1-1A* was selected for further functional analysis.

**FIGURE 5:**
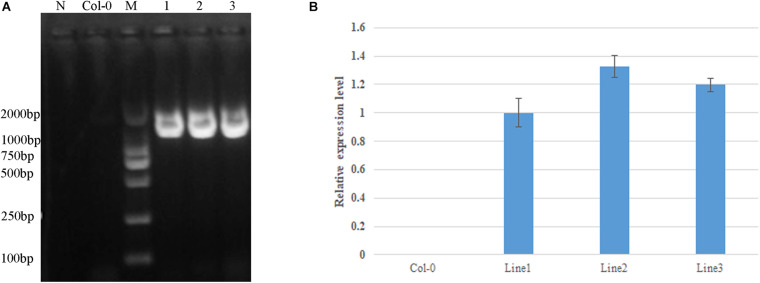
Identification of *GhDA1-1A*^*R*301*K*^ transgenic plants by PCR. **(A)** The primers used were 35S-F in the 35S promotor and GhDA1-1A-R in the *GhDA1-1A* gene (Supplementary Table 1). The “N” is the negative control without any DNA. **(B)** Relative expression level of *GhDA1-1A* in three transgenic *Arabidopsis* lines. The ΔCt value of *GhDA1-1A* in transgenic line 1 was set as the control. The data presented are the means ± SD of three biological replicates.

Based on sequence alignment, mutation site 358 in AtDA1 is equivalent to conserved amino acid 301 in the DA1-like functional domain of GhDA1-1A (Figure 4). A corresponding single-nucleotide mutation was designed as a G-to-A transition in the *GhDA1-1A* gene to cause an arginine-to-lysine change in the conserved amino acid at position 301 (Figure 4). *Arabidopsis* plants overexpressing the sequence were generated and preliminarily identified by PCR (Figure 5A). qRT-PCR was performed to further assess relative expression levels using cDNA from three different transgenic lines and WT plants as templates. A total of three lines with high expression levels were obtained and used for further studies (Figure 5B).

### Overexpression of *GhDA1-1A*^*R*301*K*^ Increases Seed Size and Seed Weight

**FIGURE 6:**
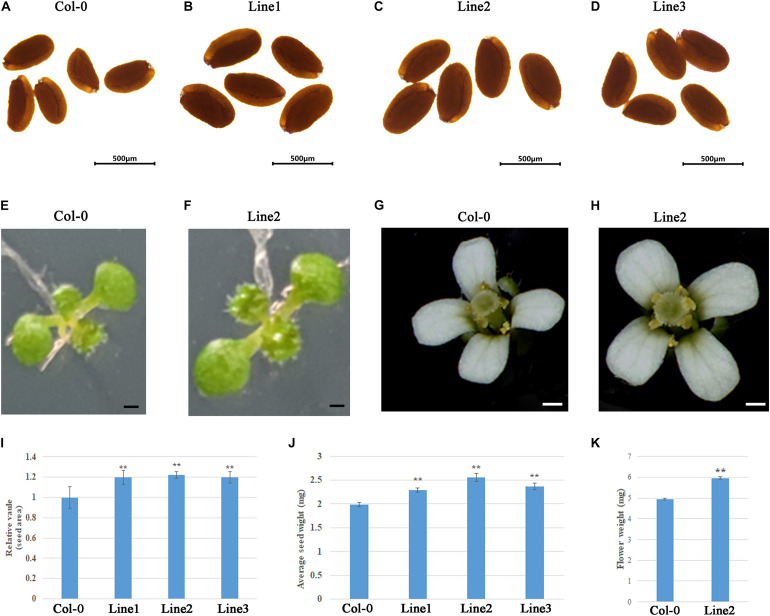
The overexpression of *GhDA1-1A*^*R*301*K*^ increases the weight and size of the seeds. **(A-D)** The seeds from transgenic plant lines were compared to seeds from CK. Bar = 500 μm. **(E)** Nine-day-old seedlings of Col-0 **(E)** and transgenic plant lines 2 **(F)**. Line2 has larger cotyledons than Col-0. **((G,H))** Flowers of Col-0 **(G)** and Line2 **(H)**. **(I)** The relative area of seeds from Col-0 and three transgenic plant lines.**(J)** Average 1000-seed weights of the CK and transgenic plant lines. Values represent the mean ± standard error from three independent samples. **(K)** Mass of five fresh flowers. **represents significant differences at the *P* < 0.01 level.

To evaluate the applicability of *GhDA1-1A*^*R*301*K*^ to transgenic breeding for seed size, we characterized the phenotypes of *GhDA1-1A*^*R*301*K*^ transgenic *Arabidopsis* at different developmental stages. The seed size of the transgenic lines was examined and was shown to be significantly increased compared to that of Col-0 (Figures 6A–D). After the seeds germinated, the cotyledon area of 9-day-old seedlings was further measured. The results showed that the seedling size of the transgenic lines was greater than that of Col-0 (Figures 6E,F). Moreover, the transgenic lines produced large flowers (Figures 6G,H), indicating that the overexpression of *GhDA1-1A^R301K^* influenced flower development and the seed mass of line 2 was increased to 128% of the Col-0 seed mass (Figures 6I–K). However, there were no differences in flowering timing, frequency, or duration (data not shown). Therefore, the overexpression of *GhDA1-1A^R301K^* increased seed size, seed weight, cotyledon size and flower size.

### GhDA1-1A Interacts With GhDA2

DA1 is a ubiquitin receptor that interacts with the E3 ubiquitin ligase DA2 to regulate seed and organ size in *Arabidopsis* and maize (Xia et al., 2013; Liu et al., 2020a). We were interested in the relationship between *GhDA1-1A* and *GhDA2* (GH_D05G3532) in *G. hirsutum.*

**FIGURE 7:**
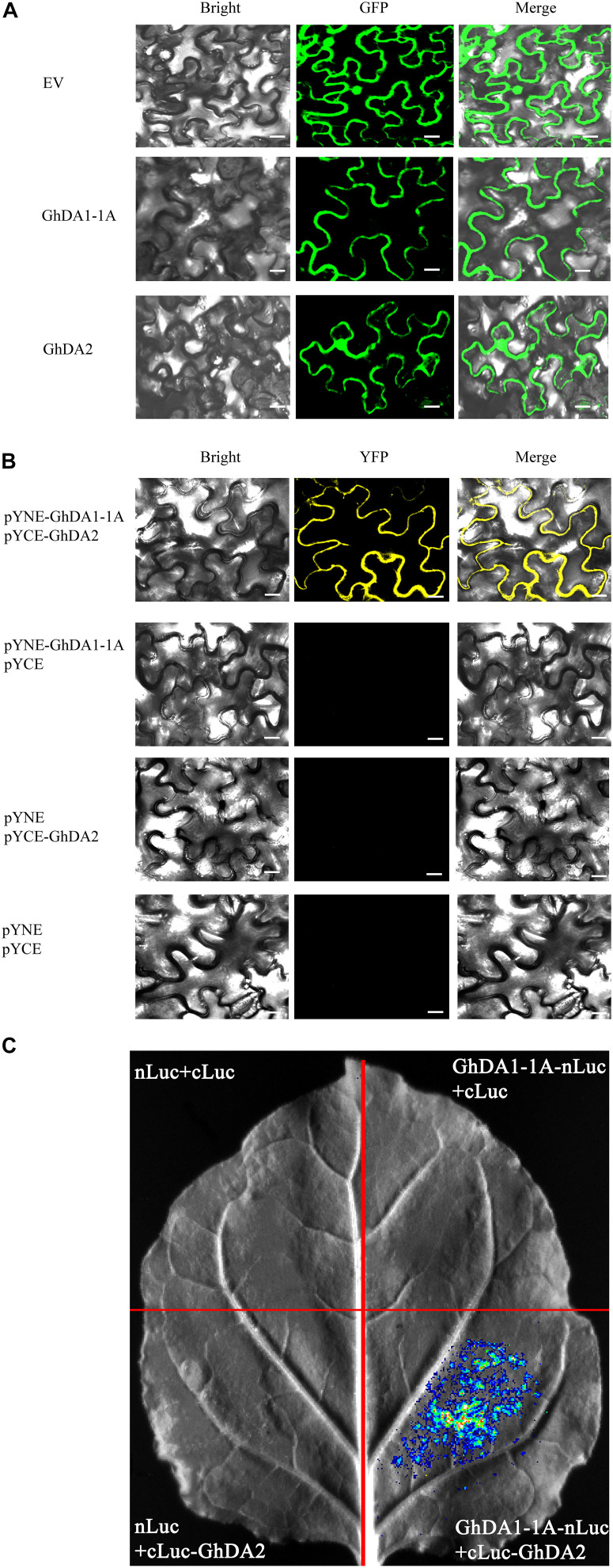
The subcellular localization and interaction of GhDA1-1A and GhDA2 **(A)** Subcellular localization of 35S-GhDA1-1A-GFP and 35S-GhDA2-GFP. **(B)** BiFC analysis of the interactions between GhDA1-1A and GhDA2. The pYNE-GhDA1-1A and the pYCE-GhDA2 were co-expressed into *N. benthamiana* leaf cells. The yellow fluorescence was detected 2 days later. **(C)** Firefly luciferase complementation imaging assay showing the interactions between GhDA1-1A and GhDA2. GhDA1-1A-nLUC and cLUC-GhDA2 were co-expressed in *N. benthamiana* leaf cells. Luciferase activity was detected 2 days after infiltration.

To determine the subcellular localization of *GhDA1-1A* and *GhDA2*, the *GhDA1-1A* and *GhDA2* construct fused with green fluorescent protein (GFP) were infiltrated into tobacco leaves, respectively. The epidermal cells of tobacco leaves were observed under a confocal microscope (Olympus). In contrast to the pattern observed in plants carrying the empty vector, the GFP signal of GhDA1-1A was found in membrane. Besides, the 35S-GhDA2-GFP signal was not only detected in the nucleus but also found in the membrane (Figure 7A).

BiFC assays were performed in tobacco leaves to test whether GhDA1-1A could interact with GhDA2. The results showed the fusion proteins of pSPYNE-GhDA1-1A and pSPYCE-GhDA2 co-localized in the membrane (Figure 7B). Then, the firefly luciferase complementation imaging assay (Chen et al., 2008) was used to provide further evidence for the interactions between GhDA1-1A and GhDA2 using agroinfiltration (Figure 7C). The results indicated that GhDA1-1A may interact with GhDA2.

## Discussion

Seed size is a key agronomic trait that strongly affects the grain yield of plants (Liu et al., 2020b). However, in cotton, fibers are the main economic product. Given that fiber cells develop from the cotton seed epidermis, seed development strongly influences fiber growth, yield, and quality (Ruan, 2013). Cotton seeds are also the sixth-largest source of vegetable oil worldwide (Liu et al., 2009). Large-seeded plants accumulate abundant nutrients to increase stress tolerance, whereas small-seeded plants flourish via dispersal and colonization (Moles et al., 2005). The mechanisms controlling seed size are thus of interest in both agriculture and biology.

In recent years, with the completion of various plant genome sequences, *DA1-like* genes in *Arabidopsis*, *Brassica napus*, *Zea mays*, and *Triticum aestivum* have been cloned and functionally verified (Li et al., 2008; Wang et al., 2017; Xie et al., 2018; Liu et al., 2020a). The overexpression of *AtDA1*^*R*358*K*^ increases seed size in *Arabidopsis* and *B. napus*. In addition, the expression of the *Zmda1* or *Zmdar1* mutant gene improves grain yield in maize. The overexpression of *TaDA1* decreases the size and weight of wheat kernels, while RNA interference (RNAi) has the opposite effect. The above results may suggest that mutation is necessary to increase seed size.

In the present study, the *DA1-like* genes of four typical *Gossypium* cotton species were identified. Three, four, seven, and seven *DA1* genes were identified in *G. raimondii*, *G. arboreum*, *G. hirsutum*, and *G. barbadense*. Previous studies have shown that DA1 proteins without the UIM and LIM domains exist in crop plants, including rice and maize (Li et al., 2008). Our studies also identified some UIM domain-lacking DA1-like genes, such as GrDA1-1, GhDA1-3D, GbDA1-3D, GaDA1-4, GhDA1-4A, and GbDA1-4A, as typical representatives of DA1-like genes.

*DA1-like* genes play important roles in increasing seed yield and biomass during plant growth and development (Li et al., 2008; Wang et al., 2017; Liu et al., 2020a). Therefore, sequence alignment was used to identify protein sequence similarities to AtDA1. All *GhDA1-like* genes were shown to be conserved, and the GhDA1-1A homolog shared 63.79% identity to AtDA1. AtDA1 and GhDA1-1A contain the same functional domains, including two UIM domains, a LIM domain, and a DA1-like functional domain, suggesting their similar functions. Furthermore, *GhDA1-1A* and *AtDA1* show similar expression patterns and are both widely expressed. Therefore, *GhDA1-1A* was cloned from *G. hirsutum* for further research. Previous studies have shown that OsDA1 is detectable around the plasma membrane of tobacco epidermal cells, while the fused TaDA1-A, -B, or -D proteins are distributed throughout the cytoplasm and nucleus (Liu et al., 2020a).

Because the overexpression of *AtDA1*^*R*358*K*^ was shown to increase seed size and seed weight (Li et al., 2008; Wang et al., 2017), the *GhDA1-1A*^*R*301*K*^ mutant was transformed into *Arabidopsis*, which increased seed size and seed weight. Moreover, the transgenic plants produced larger seedlings and larger flowers than the wild-type plants, indicating that *GhDA1-1A* plays important roles in regulating seed and organ size. Researches had showed that the DA1 can interacts with DA2 in *Arabidopsis* and maize (Xia et al., 2013; Liu et al., 2020a). However, the relationship between DA1 and DA2 was unknow in cotton. Therefore, the BiFC assay and dual-luciferase reporter assay were performed and verified that GhDA1-1A has an interaction with GhDA2, which showed that *GhDA2* may function synergistically with *GhDA1-1A* to regulate seed size in cotton.

Previous studies showed that the seedling vigor and oil content are related to seed size. Seedlings from large seeds were more vigorous than the small ones in *Brassica napus* and cotton (Elliott et al., 2008; Pahlavani et al., 2009). In addition, there was a positive correlation between oil content and seed size in cotton (Pahlavani et al., 2009; Snider et al., 2014). Cotton ovules are composed of developing seeds and fibers. Developing seeds and fibers assimilate photosynthetic products through the same ovules. However, the relationship between seed size and fiber is complex and needs to be studied further. In our future work, we will generate transgenic cotton lines to explore the relationship between seed size and fiber.

In summary, we performed a genome-wide comprehensive analysis of *DA1-like* genes in the four sequenced *Gossypium* species and identified a total of 21 *DA1-like* genes. Larger seeds and organs present potential economic value in oil crop improvement and biomass energy production (Wang et al., 2016). The overexpression of *GhDA1-1A*^*R*301*K*^ in *Arabidopsis* increased seed size and size weight, which may be useful in the improvement of seed size in cotton. Our detailed analysis of *DA1-like* genes in cotton has far-reaching significance for breeding work.

## Data Availability Statement

The datasets presented in this study can be found in online repositories. The names of the repository/repositories and accession number(s) can be found in the article/Supplementary Material.

## Author Contributions

JY and XZ directed the experiments. LH, JS, LL, YB, BJ, LW, YX, MW, and JZ participated in the study. SY conceived the study, performed the experiments, and wrote the manuscript. JY, XZ, and JZ revised the manuscript. All authors read and approved the final manuscript.

## Conflict of Interest

The authors declare that the research was conducted in the absence of any commercial or financial relationships that could be construed as a potential conflict of interest.

## Footnotes

1 http://www.arabidopsis.org

2 https://www.soybase.org

3 https://www.cottongen.org/

4 http://pfam.sanger.ac.uk/

5 http://smart.emblheidelberg.de/

6 http://web.expasy.org/

7 https://www.ncbi.nlm.nih.gov/Structure/bwrpsb/bwrpsb.cgi

8 http://bar.utoronto.ca/efp/cgi-bin/efpWeb.cgi

9 http://meme.nbcr.net/meme/

## Supplementary Material

Supplementary Figure 1 Chromosomal localization of *DA1-like* genes in four *Gossypium* species. A total of 21 *DA1-like* genes were mapped onto different chromosomes of *G. arboreum*** (A)**, *G. raimondii*** (B)**, *G. hirsutum* **(C)**, and *G. barbadense* **(D)**. The scale represents megabases (Mb).

Supplementary Figure 2 Expression level of *DA1-like* genes in *G. hirsutum* and *Arabidopsis*. **(A)** Expression patterns of 7 *GhDA1-like* genes. **(B)** Expression patterns of *AtDA1* and *AtDAR1-7* genes.

Supplementary Table 1 Primers used in this study.

Supplementary File 2 Coding sequence of *GhDA1-1A*, *GhDA1-1A*^*R301K*^ and *GhDA2*.
