# Supplementary figures and images for: Genome-Wide Analysis of DA1-Like Genes in Gossypium and Functional Characterization of GhDA1-1A Controlling Seed Size
Source: Front Plant Sci. 2021 May 20;12:647091. doi: 10.3389/fpls.2021.647091 (PMC8173226; doi:10.3389/fpls.2021.647091)

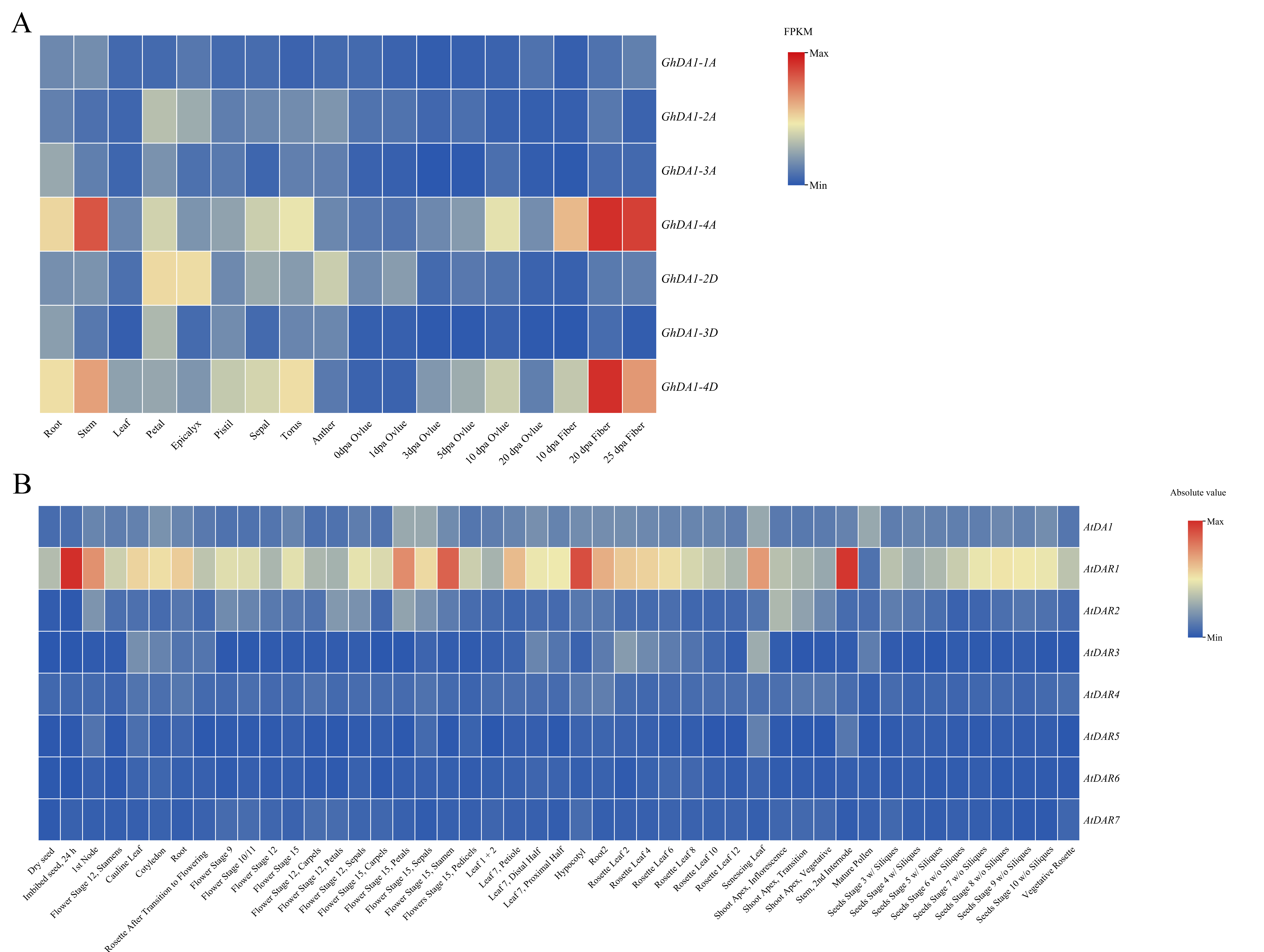

Supplement: Supplementary Figure 2 — Expression level of DA1-like genes in G. hirsutum and Arabidopsis. (A) Expression patterns of 7 GhDA1-like genes. (B) Expression patterns of AtDA1 and AtDAR1-7 genes. [file Image_2.TIF]
