# Supplementary material for: Genome-Wide Analysis of DA1-Like Genes in Gossypium and Functional Characterization of GhDA1-1A Controlling Seed Size
Source: Front Plant Sci. 2021 May 20;12:647091. doi: 10.3389/fpls.2021.647091 (PMC8173226; doi:10.3389/fpls.2021.647091)
Supplement: Supplementary Table 1 — Primers used in this study. [file Data_Sheet_1.docx]

**Additional file 1: Table S1.** Primers used in this paper.

| **Number** | **Name** | **Sequence (5′-3′)** |
| --- | --- | --- |
| 1 | CDS-GhDA1-1A-F | ATGGATTGGATTAAAAAAATT |
| 2 | CDS-GhDA1-1A-R | TCAGTAAGGAAATCTGCCGGTC |
| 3 | 35S::GhDA1-1A^R301K^-F | GGACTCTAGAGGATCCATGGATTGGATTAAAAAAATT |
| 4 | 35S::GhDA1-1A^R301K^-R | GATCGGGGAAATTCGAGCTCTCAGTAAGGAAATCTGCCGGTC |
| 5 | qRT-GhDA1-1A-F | AAAACTCAGAAGGATATTATC |
| 6 | qRT-GhDA1-1A-R | GCATTGTCAT ATTGGGGAGGAGG |
| 7 | qRT-GhDA1-2-F | CAGGATTTGTGCGGGTTGCAATACC |
| 8 | qRT-GhDA1-2-R | TCTCTATGTCTGGTAATTACCGT |
| 9 | qRT-GhDA1-3-F | ATCGTCTAAGAAAGGAGGGAG |
| 10 | qRT-GhDA1-3-R | TCAAACCTTAGACCATATTCGCC |
| 11 | qRT-GhDA1-4-F | AAGATGAGTCTGAACCGGAGGAGG |
| 12 | qRT-GhDA1-4-R | CGTGAAGAGAAAGAGGATGACC |
| 13 | At-Actin-F | AGAAACCCTCGTAGATTGGCAC |
| 14 | At-Actin-R | ACTCTCCCGCTATGTATGTCGC |
| 15 | 35S-GhDA1-1-GFP-F | GGGGCCCGGGGTCGACATGGATTGGATTAAAAAAATT |
| 16 | 35S-GhDA1-1-GFP-R | TACCGGATCCACTAGTGTAAGGAAATCTGCCGGT |
| 17 | GhDA1-1A-nLuc-F | GAGAACACGGGGGACGAGCTCATGGATTGGATTAAAAAAATT |
| 18 | GhDA1-1A-nLuc-R | CGCGTACGAGATCTGGTCGACGTAAGGAAATCTGCCGGTC |
| 19 | c-Luc-GhDA2-F | TACGCGTCCCGGGGCGGTACCATGGGTAATAAGTTGGGAAG |
| 20 | c-Luc-GhDA2-R | ACGAAAGCTCTGCAGGTCGAC CTATTGCCATGGAACTCCAGG |
| 21 | CDS-GhDA2-F | ATGGGTAATAAGTTGGGAAG |
| 22 | CDS-GhDA2-R | CTATTGCCATGGAACTCCAGG |
| 23 | mGhDA1-1Ar | GAAAGGCAGAGTCCTTTAGTCTCTGGC |
| 24 | mGhDA1-1Af | GCCAGAGACTAAAGGACTCTGCCTTTC |
