# Supplementary material for: Genome-Wide Analysis of DA1-Like Genes in Gossypium and Functional Characterization of GhDA1-1A Controlling Seed Size
Source: Front Plant Sci. 2021 May 20;12:647091. doi: 10.3389/fpls.2021.647091 (PMC8173226; doi:10.3389/fpls.2021.647091)
Supplement: Supplementary File 2 — Coding sequence of GhDA1-1A, GhDA1-1AR301K and GhDA2. [file Data_Sheet_2.docx]

**Additional file 2:**

Coding sequence of *GhDA1-1A*

1 ATGGATTGGATTAAAAAAATTTTTAAGGGCTCTGGCCAAAAAAACTCAGAAGGATATTAT

61 CATGGGGATTATGCAGAGGATCCTCAATTTTATGCACCATCTGGCACAGGGAACATGTGG

121 CAGGAGCATGAGAATGAAGAGATAGATCGAGCTATTGCCCTATCCCTTCTAGAGGAAAGT

181 CAGAAGGAGAGAAACATAATAAATGATGAATCTCAGCTTGAGGAAGATGAGCAACTTGCG

241 AGGGCTATACAAGAAAGCTTGAGGTTTGAACCTCCTCCCCAATATGACAATGCAAATACA

301 TTCCTGCCAATTCCAATCCATTTTCCGATGGGAGACAGGATTTGTGCTGGCTGCAATACT

361 GAGATTGGTCATGGAAGATTTTTAAATTGCCTCAATGCATTTTGGCATCCAGAATGTTTC

421 CATTGCCATGCTTGCAACTTTCCAATTTCTGATTATGAGTTCTCTGTGTCTGGGAATTAT

481 CGTTTCCATAAATCTTGTTATAAGGAGCGTTACCATCCAAAATGTGATGTCTGCAGCCAC

541 TTTATTCCAACAAACCCTGCTGGTCTTATTGAATATAGGGCACATCCTTTTTGGATCCAG

601 AAGTACTGCCCTTCTCATGAACATGATGGTACTCCACGATGCTGCAGCTGCGAGCGAATG

661 GAGCCACAAGACACGGGATATGTTGCCCTTGACGATGGACGGAAGCTCTGCCTTGAGTGT

721 CTAGATTCTGCAGTTATGGATACCAAGGAATGCCAACCCCTTTATCTTGATATACAAGAG

781 TTTTATGAAGGGTTAAACATGAAAGTGGAGCAGCAAGTTCCATTACTCCTGGTTGAAAGG

841 CAGGCGTTAAATGAAGCCAGAGTAGGAGAAAAGAATGGCCATTATCATATGCCAGAGACT

901 AGAGGACTCTGCCTTTCTGAAGAACAAACTGTCAGCACTATTCTAAGGCGACCAAGTTTT

961 GGGACTGGAAATCGAGCCATGGACATGATAACAGAGCCATACAAATTGACACGTAGATGT

1021 GAAGTGACTGCTATTCTCATTTTATATGGCCTGCCTAGGTTACTGACGGGGTCGATCTTA

1081 GCGCACGAGATGATGCATGCATGGATGCGACTTCAAGGTTTCCAGACTCTGAGTCAGGAC

1141 GTGGAGGAAGGTATTTGTCAGGTATTAGCGCACATGTGGATAGCTACGCAGCTCGATTCT

1201 TCTTCGAGCAGCGATGTTGCGCCGACATCATCCTCGGGTTCTAGTAGAATAAGAAAAGGC

1261 AAAAGACCCGAATTTGAGAGGAAGCTGGGTGAGTTCTTCAAGCATCAAATAGAATCAGAC

1321 ACATCGCCCGTATATGGAGATGGGTTTAGAGCAGGGAATCAGGCAGTTTATAAATATGGA

1381 CTTAGAAGAACTCTTGAACATATTCGGATGACCGGCAGATTTCCTTACTGA

Coding sequence of *GhDA1-1A^R301K^*

1 ATGGATTGGATTAAAAAAATTTTTAAGGGCTCTGGCCAAAAAAACTCAGAAGGATATTAT

61 CATGGGGATTATGCAGAGGATCCTCAATTTTATGCACCATCTGGCACAGGGAACATGTGG

121 CAGGAGCATGAGAATGAAGAGATAGATCGAGCTATTGCCCTATCCCTTCTAGAGGAAAGT

181 CAGAAGGAGAGAAACATAATAAATGATGAATCTCAGCTTGAGGAAGATGAGCAACTTGCG

241 AGGGCTATACAAGAAAGCTTGAGGTTTGAACCTCCTCCCCAATATGACAATGCAAATACA

301 TTCCTGCCAATTCCAATCCATTTTCCGATGGGAGACAGGATTTGTGCTGGCTGCAATACT

361 GAGATTGGTCATGGAAGATTTTTAAATTGCCTCAATGCATTTTGGCATCCAGAATGTTTC

421 CATTGCCATGCTTGCAACTTTCCAATTTCTGATTATGAGTTCTCTGTGTCTGGGAATTAT

481 CGTTTCCATAAATCTTGTTATAAGGAGCGTTACCATCCAAAATGTGATGTCTGCAGCCAC

541 TTTATTCCAACAAACCCTGCTGGTCTTATTGAATATAGGGCACATCCTTTTTGGATCCAG

601 AAGTACTGCCCTTCTCATGAACATGATGGTACTCCACGATGCTGCAGCTGCGAGCGAATG

661 GAGCCACAAGACACGGGATATGTTGCCCTTGACGATGGACGGAAGCTCTGCCTTGAGTGT

721 CTAGATTCTGCAGTTATGGATACCAAGGAATGCCAACCCCTTTATCTTGATATACAAGAG

781 TTTTATGAAGGGTTAAACATGAAAGTGGAGCAGCAAGTTCCATTACTCCTGGTTGAAAGG

841 CAGGCGTTAAATGAAGCCAGAGTAGGAGAAAAGAATGGCCATTATCATATGCCAGAGACT

901 AAAGGACTCTGCCTTTCTGAAGAACAAACTGTCAGCACTATTCTAAGGCGACCAAGTTTT

961 GGGACTGGAAATCGAGCCATGGACATGATAACAGAGCCATACAAATTGACACGTAGATGT

1021 GAAGTGACTGCTATTCTCATTTTATATGGCCTGCCTAGGTTACTGACGGGGTCGATCTTA

1081 GCGCACGAGATGATGCATGCATGGATGCGACTTCAAGGTTTCCAGACTCTGAGTCAGGAC

1141 GTGGAGGAAGGTATTTGTCAGGTATTAGCGCACATGTGGATAGCTACGCAGCTCGATTCT

1201 TCTTCGAGCAGCGATGTTGCGCCGACATCATCCTCGGGTTCTAGTAGAATAAGAAAAGGC

1261 AAAAGACCCGAATTTGAGAGGAAGCTGGGTGAGTTCTTCAAGCATCAAATAGAATCAGAC

1321 ACATCGCCCGTATATGGAGATGGGTTTAGAGCAGGGAATCAGGCAGTTTATAAATATGGA

1381 CTTAGAAGAACTCTTGAACATATTCGGATGACCGGCAGATTTCCTTACTGA

The red “A” represents the single-nucleotide G-to-A transition.

Coding sequence of *GhDA2*

1 ATGGGTAATAAGTTGGGAAGGAGAAGGCAAGTGGTGGACGAGAAGTATACGCGCCCCCAA

61 GGGTTGTATGTTCATAAAGATGTGGATGTTAAGAAGCTGAGAAAACTGATACTTGAATCG

121 AAGCTTGCTCCATGTTACCCTGGCAATGAAGAGTGCTGTTATGATCTTGAAGAATGCCCA

181 ATTTGCTTTTTGTATTACCCGAGTCTCAACAGATCAAGATGTTGCATGAAAAGTATTTGC

241 ACAGAGTGTTTTCTACAGATGAAGAATCCAAACTCGACCCGTCCTACCCAGTGTCCTTTC

301 TGCAAAACCTCAAACTACGCTGTGGAGTACCGAGGTGTGAAAACAAAGGAGGAAAAAGGG

361 ATCGAGCAAATTGAAGAACAACGTGTCATAGAAGCACAAATTAGAATGAGGCAGCAGGAA

421 CTTCAGGATGACGAAGAGAGAATGCAGAAAAGACAAGAATTCAGTTCTTCAAGCACCGCT

481 GTTTCACCGGGGGAAGTTCAATACGGTACAGCTGCTGCTCAATCCTCTGTTGAGGAGGAA

541 CTAGTTTCTTCTCAAGATTCGCAGGCTGCCATGATGGTTCAACAACCATCACATCCTAGG

601 ACAAACAGGGATGATGAGTTTGACGTAGATCTGGAGGAAATAATGGTCATGGAAGCAATT

661 TGGCAGTCGATTCAGGAGAACAGCAGACACAGAAAGTCTAACAATGGAGATGCTGCTTCT

721 TCAGTACATGTTTCAGTAGATCGCTATGTCTCACCAGCTATGGCCACAGTGGCCGGTTCA

781 TCATCATCATCATCATCATCATCTCCTTCTGGTGGTTTTGCTTGTGCAGTAGCTGCCCTT

841 GCTGAGCGTCAGCAGATCAGTGGAGAATCTTCTCTTGACTACAATGGAAATATACCACCG

901 TTCAATATGCTTCCTGGCAGCAGCAGGTTTTATAACAGGTTGGACCCAGTTTCCGAGAAT

961 CGTCCTGCAGAGAGCCCGGTTGACATGCCAACTGGTGGTCTGATGACACCTTCAAGAGAT

1021 GAAGGGGAATGGGGAGTAGATTTTGGATCGGAGGTGGCTGAAGCAGGGACTAGCTACGCA

1081 AGTCCTGATGTTACAGAAGATATAGGCGGGATCTCAACAATACCACAACAGGATGAAATA

1141 AGGGGTAGCTTTCTAAATGTGCCCCGACCCATTGTTCCGGAAAGTTATGAAGAGCAGATG

1201 ATGCTGGCTATGGCTGTATCTCTGTCTGAAGCTAAAGCTATGACAAGTAACCCTGGAGTT

1261 CCATGGCAATAG
